# An integrative, genomic, transcriptomic and network-assisted study to identify genes associated with human cleft lip with or without cleft palate

**DOI:** 10.1186/s12920-020-0675-4

**Published:** 2020-04-03

**Authors:** Fangfang Yan, Yulin Dai, Junichi Iwata, Zhongming Zhao, Peilin Jia

**Affiliations:** 1 Center for Precision Health, School of Biomedical Informatics, The University of Texas Health Science Center at Houston, 7000 Fannin St. Suite 600, Houston, TX 77030 USA; 2 Department of Diagnostic and Biomedical Sciences, School of Dentistry, The University of Texas Health Science Center at Houston, Houston, TX 77054 USA; 3 Center for Craniofacial Research, The University of Texas Health Science Center at Houston, Houston, TX 77054 USA; 4 Human Genetics Center, School of Public Health, The University of Texas Health Science Center at Houston, Houston, TX 77030 USA; 5 Department of Biomedical Informatics, Vanderbilt University Medical Center, Nashville, TN 37203 USA

**Keywords:** Cleft lip, Cleft palate, Dense module search, Genome-wide association studies (GWAS), Network

## Abstract

**Background:**

Cleft lip with or without cleft palate (CL/P) is one of the most common congenital human birth defects. A combination of genetic and epidemiology studies has contributed to a better knowledge of CL/P-associated candidate genes and environmental risk factors. However, the etiology of CL/P remains not fully understood. In this study, to identify new CL/P-associated genes, we conducted an integrative analysis using our in-house network tools, dmGWAS [dense module search for Genome-Wide Association Studies (GWAS)] and EW_dmGWAS (Edge-Weighted dmGWAS), in a combination with GWAS data, the human protein-protein interaction (PPI) network, and differential gene expression profiles.

**Results:**

A total of 87 genes were consistently detected in both European and Asian ancestries in dmGWAS. There were 31.0% (27/87) showed nominal significance with CL/P (gene-based *p* < 0.05), with three genes showing strong association signals, including *KIAA1598*, *GPR183*, and *ZMYND11* (*p* < 1 × 10^− 3^). In EW_dmGWAS, we identified 253 and 245 module genes associated with CL/P for European ancestry and the Asian ancestry, respectively. Functional enrichment analysis demonstrated that these genes were involved in cell adhesion, protein localization to the plasma membrane, the regulation of the apoptotic signaling pathway, and other pathological conditions. A small proportion of genes (5.1% for European ancestry; 2.4% for Asian ancestry) had prior evidence in CL/P as annotated in CleftGeneDB database. Our analysis highlighted nine novel CL/P candidate genes (*BRD1*, *CREBBP*, *CSK*, *DNM1L, LOR*, *PTPN18*, *SND1, TGS1*, and *VIM*) and 17 previously reported genes in the top modules*.*

**Conclusions:**

The genes identified through superimposing GWAS signals and differential gene expression profiles onto human PPI network, as well as their functional features, helped our understanding of the etiology of CL/P. Our multi-omics integrative analyses revealed nine novel candidate genes involved in CL/P.

## Background

Cleft lip with or without cleft palate (CL/P) is one of the most common congenital human birth defects [1, 2]. The prevalence of CL/P ranges from 7.75 to 10.89 per 10,000 live births, with ethnic, racial, and geographic variation [1, 3]. Multiple interventions are required in treatments for individuals with CL/P, such as medical, surgical, speech, and behavioral, imposing an economic burden to the family [4, 5]. Approximately 70% of CL/P cases occur as sporadic (nonsyndromic) and the remaining 30% are a part of syndromic phenotypes [6]. The syndromic cases can be Mendelian traits or cases with chromosome aberrations [5]. In addition to genetic variants, environmental factors play a crucial role in nonsyndromic CL/P [7]. Epidemiologic studies have identified several environmental risk factors of CL/P, including alcohol abuse, certain drug exposure, nutrition deficiency, and smoking [8].

Efforts have been made in uncovering the complex etiology of CL/P by using various genetic approaches, such as linkage analyses, direct sequencing of individuals with CL/P, association studies, and animal model studies. For example, a genome-wide linkage study in the Malay population identified several CL/P candidate genes, including *LPHN2*, *PVRL3*, and *SATB2* [9]. Whole exome sequencing revealed a change in copy number of *ADH7* and *AHR* in nonsyndromic CL/P [10]. Genome-wide association studies (GWAS) of CL/P have made major advances in the identification of candidate genes and *loci* in CL/P. A case-parent trio GWA study consisting of cases from the European and Asian populations showed variants near *MAFB* and *ABCA4* to be significantly associated with CL/P [2]. Variants near *CTNNA2* and *SULT2A1* were discovered in another GWA study conducted in the African American population [11].

While new genetic mutations have been identified in CL/P cases, the etiology of CL/P remains elusive. Especially, the variants discovered by GWAS mostly reside in non-coding genomic regions, making it difficult to explore the functional roles of these variants in the pathogenesis of CL/P [12]. Although some gene-set based methods such as Gene Set Enrichment Analysis (GSEA) have helped identify the combined effect of multiple gene markers, these methods are limited to pre-defined knowledge and suffer from the incompleteness of functional database annotations [13, 14]. dmGWAS (dense module search for Genome-Wide Association Studies) is a network-assisted approach to identifying disease-associated signals which were missed by the stringent genome-wide significance level (e.g., 5 × 10^− 8^). By superimposing GWAS signals onto the human reference protein-protein interaction (PPI) networks, dmGWAS searches for dense modules by implementing a greedy searching method in the node-weighted PPI network [15]. dmGWAS has been successfully applied in cancer, pediatric stroke, chronic obstructive pulmonary disease, schizophrenia, and osteosarcoma [16–18], among others [13, 15]. EW_dmGWAS (Edge-Weighted dense module search for Genome-Wide Association Studies) is the upgraded algorithm of dmGWAS, which integrates not only GWAS signals but also gene expression profiles in order to identify dense modules in node-weighted and edge-weighted PPI network [19]. The importance of differential gene expression has been demonstrated in a study of hepatocellular carcinoma because it represents disease-associated transcriptional information [20]. Combination of gene expression profiles and PPI network could outperform traditional analysis in uncovering the mechanisms of disease. EW_dmGWAS utilizes differential gene expression to infer edge weights. When evaluating a module, EW_dmGWAS considers both the node weight (GWAS signals) and the edge weight (by comparing disease-control expression profiles or by using disease-relevant expression profiles) to calculate the module score. Thus, the dense modules from EW_dmGWAS are expected to be enriched in a disease-relevant context. For example, an integrative analysis study of alcohol-use disorders demonstrated the importance of EW_dmGWAS in search of the possible molecular mechanisms involved in alcohol-use disorders [21].

In this study, to identify new and consistent genetic signals in CL/P, we conducted dmGWAS and EW_dmGWAS. We collected two GWAS datasets for CL/P and used them as discovery and evaluation datasets, respectively. We focused on consistent signals from different populations. By adding differential gene expression profile, EW_dmGWAS was utilized to generate node- and edge-weighted networks for each population. The genes residing in the modules identified through dmGWAS and EW_dmGWAS were evaluated through functional enrichment analysis. Such results are more likely reliable than using a single GWAS dataset or only the GWAS data.

## Results

### CL/P candidate genes and subnetworks for European and Asian ancestry

**Fig. 1:**
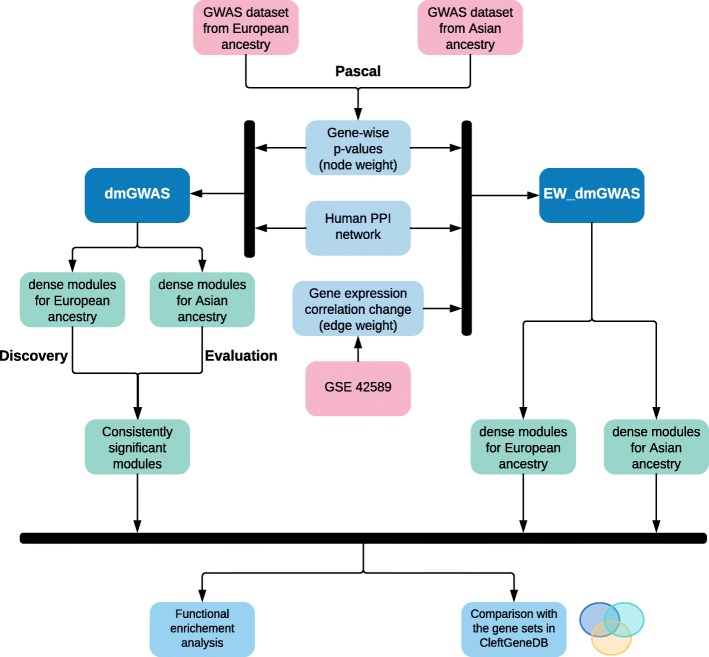
Workflow of integrative, genomic, transcriptomic, and network-assisted analyses to identify candidate genes for cleft lip with or without cleft palate in two human populations

The details of the data, process, and analytical methods are provided in the Additional files. Figure 1 illustrates our data analysis workflow. The Manhattan plots of gene-level *p*-values were displayed for European and Asian ancestry samples respectively in Additional file 1: Figure S1. In this study, we used the number of genes instead of the number of SNPs to adjust gene-level p-values and set the genome-wide significance as *p* = 0.05/ (number of genes) = 2.3 × 10^− 6^ following the stringent Bonferroni multiple test correction. This led to the significance line in Additional file 1: Figure S1 as y = −log_10_ (0.05/number of genes) = 5.63 (i.e., the horizontal line in blue). Five significant genes were notably higher than other genes in the Asian GWAS data, as shown in the Manhattan plot (Additional file 1: Figure S1B). These genes are *DIEXF* (*p* = 1.36 × 10^− 8^), *MAFB* (*p* = 3.14 × 10^− 8^), *C1orf74* (*p =* 2.47 × 10^− 7^), *IRF6* (*p* = 3.66 × 10^− 7^), and *TRAF3IP3* (*p* = 5.98 × 10^− 7^). Because such genes with extraordinary *p*-values would have overwhelmed the resultant modules should they exist in the reference network (too strong node weight), we excluded these genes from further analyses [13], and only reported here as individual gene results. Indeed, when we used all genes for dmGWAS analysis, nearly all candidate modules contained the gene *MAFB* (data not shown). Hence, we manually adjusted the p-values of these five genes to *p* = 6.39 × 10^− 5^, which was the lowest p-value after excluding the five genes.

By using the dense module search method of dmGWAS, we identified a total of 7463 modules from the European GWAS dataset containing 8122 unique genes and 7440 modules from the Asian GWAS dataset containing 8070 unique genes. There were 8042 genes overlapped between the modules of these two GWAS datasets (European 99.02%; Asian 99.65%). In the European GWAS dataset, the average module size was 13.73 ± 1.90 (range: 5–23), with an average module score of 6.22 (range: 1.33–7.00, standard deviation: 0.31). In the Asian dataset, the average module size was 13.49 ± 1.86 (range: 5–23) and the average module score (Z_n_) was 6.88 in Asian GWAS dataset (range: 1.47–8.04, standard deviation: 0.42).

#### Subnetworks for European ancestry

The module with the highest score consisted of 12 genes (*BIRC8, BRF1, CASP9, CDK2, ERBB3, PA2G4, POLL, PPM1B, RB1, SMARCA4,* and *ZMYND11*) (Additional file 2: Figure S2A)*.* Interestingly, not all genes in this module had strong association signals. For example, *RB1* had a relatively weak association signal (*p* = 0.08); however, it directly or indirectly interacted with many other genes that had strong association *p*-values, such as *CDK2* (*p* = 8.7 × 10^− 3^), *PA2G4* (*p* = 0.01), *ERBB3* (*p* = 6.5 × 10^− 3^), *BIRC8* (*p* = 9.3 × 10^− 4^), and *ZMYND11* (*p* = 5.7 × 10^− 4^). We further examined the top 10 modules and identified 57 CL/P candidate genes (Additional file 2: Figure S2B). There were 35 (61.4%) genes showing nominal significant association signals (defined as *p* < 0.05 without multiple testing correction). The top 50 modules from the European GWAS dataset formed a final subnetwork consisting of 133 non-redundant genes. Among these genes, 48.1% (64/133) showed nominal significance with CL/P (gene-based p < 0.05). Notably, there were a total of 37 genes with *p* < 1 × 10^− 3^ in the network and 6 were included in our final subnetwork: *BIRC8, CAND1*, *GPR183*, *TMEM11*, *TRPM2*, and *ZMYND11* (p < 1 × 10^− 3^). Collectively, these results implied the strong ability of dmGWAS in finding disease-associated genes which had a weak or moderate association signal.

Next, gene set enrichment analysis was conducted to functionally categorize these candidate genes. After clustering the gene sets using the affinity propagation method, eight Gene Ontology (GO) terms were enriched that highlighted the importance of immune response-regulating cell surface receptor signaling pathway (15 module genes, adjusted *p*-value = 9 × 10^− 5^), cell membrane protein (9 module genes, adjusted p-value = 1.1 × 10^− 3^), and apoptotic pathway (14 module genes, adjusted p-value = 1.1 × 10^− 4^). Among the KEGG pathways, we observed the enrichment in virus infection (18 genes, adjusted p-value = 1.5 × 10^− 8^) and ErbB signaling pathway (10 genes, adjusted p-value = 3 × 10^− 5^). These findings suggested that cell adhesion, viral infection and ErbB signaling pathway may play substantial roles in palate formation.

#### Subnetworks for Asian ancestry

The top 1 module consisted of 13 genes (*ARHGAP29, CTNNB1, EPHA7, GRIA2, JUN, MAFB, MLLT4, MYOD1, OGG1, PICK1, PITX2, PRKCA,* and *RAP2A*) (Additional file 2: Figure S2C)*.* Importantly, *MAFB* was reported in the original GWA study because the Single Nucleotide Polymorphisms (SNPs) near this gene presented the strongest association signals [11]. The top 10 modules consisted of 35 CL/P-associated genes, with 51.4% (18/35) showing significant association signals (Additional file 2: Figure S2D). The top 50 candidate modules with the highest module scores were merged to construct the final subnetwork, including 113 candidate genes. Among these 113 genes, 45.1% (51/113) had nominal significant *p*-values (defined as *p* < 0.05 without multiple testing correction) and seven genes (0.6%) with strong GWAS signals; *ARHGAP29*, *MAFB*, *CAPN3*, *DNMT3B*, *GMEB1, OGG1*, and *PITX2* (*p* < 1 × 10^− 3^). Notably, there were 9 genes overlapped between 133 genes for European population and 113 candidate genes for Asian population: *ACTN1, AKT1, CASP9, CDK2, COIL, HDAC1, MAPK3, PRPF40A,* and *TTN*.

Enrichment analysis of these 113 genes indicated the importance of cell membrane protein (13 module genes, adjusted *p*-value = 2.4 × 10^− 5^), cell differentiation (12 module genes, adjusted p-value = 6 × 10^− 3^), and apoptotic signaling during palate formation (6 module genes, adjusted p-value = 0.02). The KEGG pathway analysis suggested that these genes were involved in Focal adhesion (13 module genes, adjusted p-value = 2.1 × 10^− 5^), HIV infection (11 module genes, adjusted p-value = 2.3 × 10^− 3^), and ErbB signaling pathway (7 module genes, adjusted p-value = 6.4 × 10^− 3^), which was consistent with the results from European ancestry.

#### Consistent association signals identified by dualEval

**Fig. 2:**
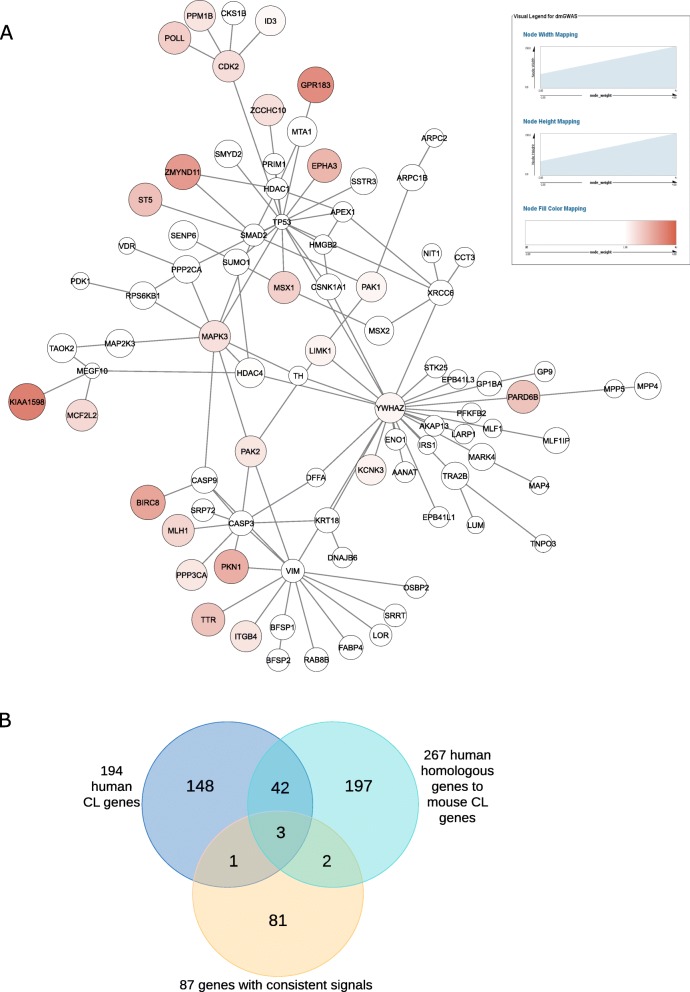
Consistent association signals identified by dmGWAS. (A) The subnetwork of all significant modules in two populations visualized by Cytoscape showing the consistent signals. Each circle represents a gene. For the genes with *p*-value ≥0.05 (insignificant), the color of the circle is set to white. For genes with p-value < 0.05, the area and color of the circle is proportional to gene’s weight. The stronger the color (red) is, the smaller the p-value is, and the stronger weight the gene is assigned. (B) A Venn diagram shows the overlap of genes from three sources. The yellow circle represents the 87 genes with consistent signals. The blue circle represents 194 human CL/P associated genes in the CleftGeneDB database. The green circle represents the 267 human genes homologous to mouse mutation genes in the CleftGeneDB database

By treating European as a discovery set and Asian as an evaluation set, we identified a total of 40 modules consisting of 87 genes with consistent signals in the PPI subnetwork (Fig. 2a). Among these 87 genes, 31.0% (27/87) showed significant association signals (*p* < 0.05). Three genes showed strong association signals, including *KIAA1598*, *GPR183*, and *ZMYND11* (*p* < 1 × 10^− 3^). The module size ranged from 12 to 13. The normalized module score ranged from 6.71 to 6.93 in the European (discovery) dataset and from 1.68 to 2.70 in the Asian (evaluation) dataset. Four of these module genes (4.6%) were overlapped with the genes collected in the CleftGeneDB database [22], a database with manual curation of CL/P genes (see Methods): *MSX1*, *MSX2*, *SMAD2*, and *SUMO1* (Fig. 2b).

These 87 genes were enriched in six GO Biological Process (BP) terms (i.e., pathways), including cellular response to drug (12 genes, adjusted *p*-value = 7.2 × 10^− 4^), regulation of binding (11 genes, adjusted p-value = 9.8 × 10^− 3^), response to steroid hormone (11 genes, adjusted p-value = 0.01), Fc receptor signaling pathway (7 genes, adjusted p-value = 0.017), intermediate filament cytoskeleton organization (5 genes, adjusted p-value = 0.022), and intermediate filament-based process (5 genes, adjusted p-value = 0.024) (Table 1). Enriched KEGG pathways suggested the potential contribution of viral infection to CL/P: human papillomavirus infection (12 genes, adjusted p-value = 4.2 × 10^− 3^) and human immunodeficiency virus 1 infection (9 genes, adjusted p-value = 0.014) (Table 1). Interestingly, as shown in GO BP terms, Fc gamma receptor relevant pathway was also identified in KEGG pathway analysis (Fc gamma R-mediated phagocytosis, 6 genes, adjusted p-value = 0.027).

**Table 1:** Pathways enriched in the dmGWAS module genes (discovery: European dataset, evaluation: Asian dataset)

Pathway ID	Description	Pathway size^*^	# informative genes^$^	Adjusted p-value^#^
*GO Biological Process*				
GO:0035690	Cellular response to drug	349	12	7.2 × 10^−4^
GO:0051098	Regulation of binding	367	11	9.8 × 10^− 3^
GO:0048545	Response to steroid hormone	388	11	0.017
GO:0038093	Fc receptor signaling pathway	127	7	0.017
GO:0045104	Intermediate filament cytoskeleton organization	48	5	0.022
GO:0045103	Intermediate filament-based process	49	5	0.024
*KEGG pathway*				
hsa05210	Colorectal cancer	86	7	1.7 × 10^−3^
hsa05165	Human papillomavirus infection	339	12	4.2 × 10^−3^
hsa05170	Human immunodeficiency virus 1 infection	212	9	0.014
hsa05014	Amyotrophic lateral sclerosis (ALS)	51	5	0.016
hsa04360	Axon guidance	175	8	0.024
hsa04666	Fc gamma R-mediated phagocytosis	91	6	0.027

^*^Pathway size: the total number of genes in the GO term or KEGG pathway

^**$**^The observed number of genes from the module gene list

^#^Bonferroni method was used to adjust *p*-value

### CL/P associated node- and edge-weighted PPI network

#### Subnetworks for European ancestry

**Fig. 3:**
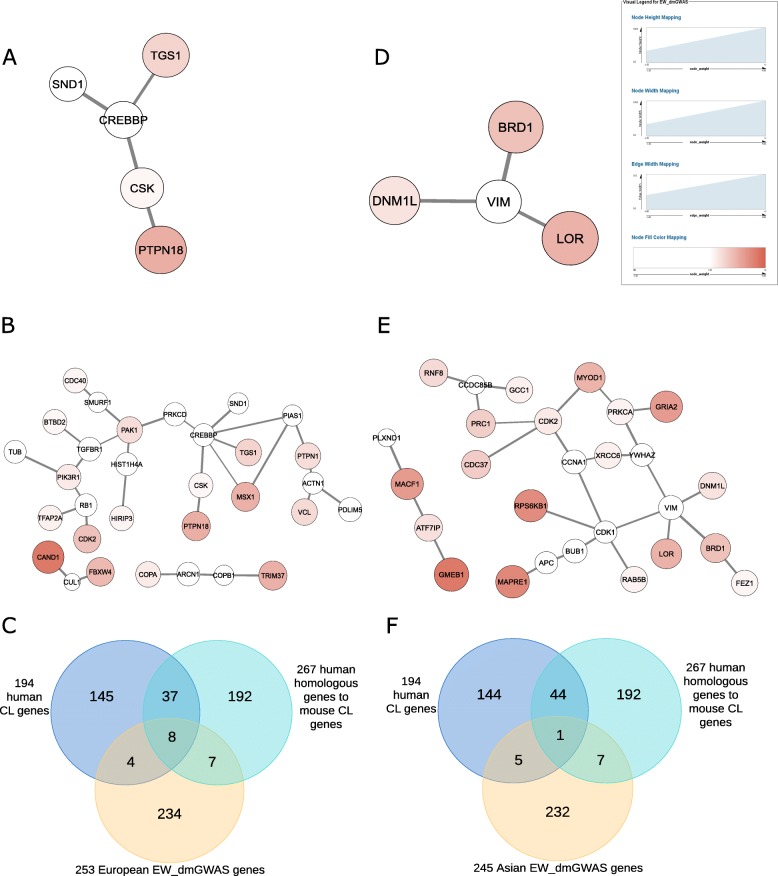
Subnetworks of module genes identified by EW_dmGWAS. (A) The top 1 network module from the data in European ancestry. Each circle represents a gene. For the genes with p-value ≥0.05 (insignificant), the color of the circle is set to white. For genes with p-value < 0.05, the area and color of the circle is proportional to gene’s weight. The stronger the color (red) is, the smaller the p-value is, and the stronger weight the gene is assigned. (B) A merged subnetwork of top 10 network modules from the data in European ancestry. (C) A Venn diagram showing the intersection of the 253 genes for European ancestry with the two gene sets in the CleftGeneDB database. (D) The top 1 module from the data in Asian ancestry. (E) A merged subnetwork of top 10 modules from the data in Asian ancestry. (F) A Venn diagram showing the intersection of the 245 genes for Asian ancestry with the two gene sets in CleftGeneDB database

Using the European GWAS data, we identified 121 modules (out of 129 modules) consisting of 253 genes (p_perm_ < 0.05) using EW_dmGWAS. Among these 253 genes, 54.2% (137/253) exhibited significant association signals with CL/P (gene-based *p* < 0.05). The module size ranged from 2 to 5. The module score ranged from 2.48 to 8.75 with a standard deviation of 1.30. The top 1 module consisted of five genes (*CREBBP*, *CSK*, *PTPN18*, *SND1,* and *TGS1*) (Fig. 3a). There were 31 genes in the top 10 modules (Fig. 3b). There were 42 genes overlapped between these 253 genes generated by EW_dmGWAS and the gene set identified by dmGWAS. In addition, 36.76% (93/253) had differential gene expression when compared the cases with the controls (p < 0.05), with expression level of 13 genes showing more than two-fold change (absolute value of log_2_FC > 1; FC: fold change). Comparison with the 194 human CL/P-associated genes in the CleftGeneDB database revealed that 12 genes (*BCL2, DVL2, FGFR1, MSX1, MSX2, SPRY2, SUMO1, TFAP2A, TGFBR1, TPM1, TP63,* and *VCL*; 5.1%) had been previously studied in CL/P (Fig. 3c).

Enrichment analysis of GO BP terms suggested that these genes were involved in cell structure and cell membrane relevant process (Table 2) [e.g. regulation of protein localization to membrane (15 module genes, adjusted *p*-value = 2.3 × 10^− 4^), regulation of actin cytoskeleton organization (18 module genes, adjusted p-value = 5.0 × 10^− 3^), immune response-regulating cell surface receptor signaling pathway (23 module genes, adjusted p-value = 2.3 × 10^− 6^)]. The KEGG pathways enrichment analysis showed that these genes were related to prostate cancer (14 module genes, adjusted p-value = 2.8 × 10^− 6^), renal cell carcinoma (11 module genes, adjusted p-value = 4.1 × 10^− 5^), and glioma (8 module genes, adjusted p-value = 0.03). Additionally, these genes were enriched in Fc gamma R-mediated phagocytosis (13 module genes, adjusted p-value = 1.1 × 10^− 5^) and B cell receptor signaling pathway (8 module genes, adjusted p-value = 0.030), suggesting the contribution of the immune response to CL/P.

**Table 2:** Pathways enriched in EW_dmGWAS module genes (European dataset)

Pathway ID	Pathway name	Pathway size^*^	# informative genes^$^	Adjusted p-value^#^
*GO Biological Process*				
GO:0002768	Immune response-regulating cell surface receptor signaling pathway	330	23	2.3 × 10^−6^
GO:0003012	Muscle system process	423	25	1.2 × 10^− 5^
GO:1905475	Regulation of protein localization to membrane	177	15	2.3 × 10^−4^
GO:0030335	Positive regulation of cell migration	472	24	4.5 × 10^− 4^
GO:0022898	Regulation of transmembrane transporter activity	235	16	1.7 × 10^−3^
GO:0090068	Positive regulation of cell cycle process	270	17	2.1 × 10^−3^
GO:0043433	Negative regulation of DNA-binding transcription factor activity	155	13	2.1 × 10^−3^
GO:0060627	Regulation of vesicle-mediated transport	499	23	4.8 × 10^−3^
GO:0032956	Regulation of actin cytoskeleton organization	320	18	5.0 × 10^−3^
GO:0010975	Regulation of neuron projection development	475	22	7.8 × 10^−3^
GO:0002433	Immune response-regulating cell surface receptor signaling pathway involved in phagocytosis	76	9	8.0 × 10^−3^
*KEGG pathway*				
hsa05215	Prostate cancer	97	14	2.8 × 10^−6^
hsa04666	Fc gamma R-mediated phagocytosis	91	13	1.1 × 10^−5^
hsa05211	Renal cell carcinoma	69	11	4.1 × 10^−5^
hsa04662	B cell receptor signaling pathway	71	8	0.030
hsa05214	Glioma	71	8	0.030

^*^Pathway size: the total number of genes in the GO term or KEGG pathway

^**$**^The observed number of genes from the module gene list

^#^Bonferroni method was used to adjust p-value

#### Subnetworks for Asian ancestry

We identified 132 modules in total with 119 modules (p_perm_ < 0.05) containing 245 genes for Asian ancestry. The module size ranged from 2 to 4. The module score ranged from 2.46 to 8.35 with a standard deviation of 1.37. Comparing with the 113 genes generated by dmGWAS for Asian ancestry, 36 genes overlapped. There were 18 genes overlapped between the 245 genes for the Asian population and the 253 genes for the European population based on EW_dmGWAS. Among the 245 genes, 42.04% (103/245) genes were differentially expressed in cases versus controls (*p* < 0.05). The expression level of 12 genes (12/245 = 4.90%) had two-fold change in cases versus controls (absolute value of log_2_FC > 1). The best module contained four genes (*BRD1*, *DNM1L, LOR*, and *VIM*) (Fig. 3d). We identified 27 genes from the top 10 modules (Fig. 3e). The intersection between these 245 genes and gene sets in the CleftGeneDB was shown in Fig. 3f. According to the Venn diagram, six genes (*BCL3*, *MSX1*, *PAX6*, *PVR, RFC1*, and *SEC16A*; 2.4%) have been reported in previous publications (Fig. 3f). A further comparison was conducted between these genes and the gene sets. We found that seven genes (*CTNNB1*, *DNMT3B*, *GRB2*, *ITGAV*, *LIMS1, PTPN11*, and *TP53*) were overlapped. Notably, none of the four genes in the best module (*BRD1*, *DNM1L, LOR*, and *VIM*) were overlapped with the CleftGeneDB gene set, suggesting that these genes has not yet been studied as CL/P candidate genes.

Functional analysis indicated that the module genes were related to cell structure and cell response to the stimulus (Table 3). Enriched KEGG pathways were mostly related to virus infection (e.g. viral carcinogenesis (22 genes, adjusted *p*-value = 2.6 × 10^− 7^) and human papillomavirus infection (26 genes, adjusted p-value = 1.4 × 10^− 5^). Apoptosis (6 genes, adjusted p-value = 0.023) was also enriched (Table 3).

**Table 3:** Pathways enriched in the EW_dmGWAS module genes (Asian dataset)

Pathway ID	Pathway name	Pathway size^*^	# informative genes^$^	Adjusted p-value^#^
*GO Biological Process*				
GO:2001233	Regulation of apoptotic signaling pathway	385	26	1.9 × 10^−7^
GO:0043062	Extracellular structure organization	400	23	6.0 × 10^−5^
GO:0043254	Regulation of protein complex assembly	447	24	1.1 × 10^−4^
GO:0007409	Axonogenesis	449	24	1.2 × 10^−4^
GO:1901653	Cellular response to peptide	358	21	2.0 × 10^−4^
GO:0031589	Cell-substrate adhesion	332	20	2.7 × 10^−4^
GO:0060627	Regulation of vesicle-mediated transport	499	24	8.5 × 10^−4^
GO:0002237	Response to molecule of bacterial origin	330	19	1.2 × 10^−3^
GO:0050730	Regulation of peptidyl-tyrosine phosphorylation	244	16	2.1 × 10^−3^
GO:0002521	Leukocyte differentiation	496	23	3.0 × 10^−3^
GO:0034330	Cell junction organization	285	17	3.3 × 10^−3^
GO:0006914	Autophagy	473	22	5.1 × 10^−3^
GO:0051123	RNA polymerase II preinitiation complex assembly	25	6	6.1 × 10^−3^
GO:0038179	Neurotrophin signaling pathway	39	7	6.1 × 10^−3^
GO:0070482	Response to oxygen levels	337	18	7.7 × 10^−3^
*KEGG pathway*				
hsa05203	Viral carcinogenesis	201	22	2.6 × 10^−7^
hsa05165	Human papillomavirus infection	339	26	1.4 × 10^−5^
hsa04728	Dopaminergic synapse	131	12	0.014
hsa04215	Apoptosis	32	6	0.023

^*^Pathway size: the total number of genes in the GO term or KEGG pathway

^**$**^The observed number of genes from the module gene list

^#^Bonferroni method was used to adjust p-value

## Discussion

In this study, we carried out the network analyses for CL/P GWAS datasets from European and Asian ancestries by using both dmGWAS and EW_dmGWAS tools. Our unique analysis design (two GWAS datasets, two network module analyses, dual evaluation, two populations, and multi-omics analysis) will likely reveal more reproducible and consistent genetic association signals and their potential function in CL/P. To our best knowledge, this comprehensive network analysis of GWAS pipeline has not been introduced in any literature. We identified 253 genes of interest for CL/P pathogenesis in the European population and 245 genes in the Asian population by EW_dmGWAS. Of note, our study is the first time to detect CL/P-associated genes in one population dataset, evaluating the signals in another population dataset. Our analysis highlighted 17 genes that are known to be associated with CL/P, such as *FGFR1*, *MSX*1, *and TFAP2A*. The protein encoded by *MSX1* gene functions as transcription repressor during embryogenesis, which plays a substantial role in craniofacial development. Both the mouse model study [23] and association studies [24, 25] confirmed the contributions of *MSX1* gene to the risk of CL/P. The *FGFR1* gene encodes the fibroblast growth factor receptor. Gene *TFAP2A* encodes transforming growth factor beta receptor. A previous association study of Italian population provided evidence for the involvement of *TFAP2A* in CL/P [26].

In addition, the best module with the most significant score contained 5 genes for European dataset and 4 genes for Asian dataset. These nine unique genes are considered as new CL/P candidate genes since they are not in the CleftGeneDB database (*BRD1*, *CREBBP*, *CSK*, *DNM1L, LOR*, *PTPN18*, *SND1, TGS1,* and *VIM*) (Fig. 3a, d). A lot of supporting evidence exist regarding the association of these nine genes and CL/P. For example, the intermediate filament protein encoded by the *VIM* gene plays an important role in the formation of cytoskeleton and stabilization of cytoskeletal interactions. The mutations in the *LOR* gene are associated with Vohwinkel’s syndrome, an inherited skin disease. The *BRD1* gene may take effect in gene activation by interacting with DNA. The *DNM1L* gene is responsible for cell division and the apoptotic process. Of interest, *VIM* was initially suspected as a CL/P candidate since it maps near to the translocation breakpoint of two CL/P individuals [27], yet the association was excluded in a subsequent linkage analysis [28]. Consistent with these findings, the *VIM* gene in our study also showed a weak association signal (*p* = 0.06). Thus, our study suggests the contribution of these genes to CL/P when taking PPI networks and gene expression correlation change into consideration.

Enrichment analysis revealed that gene sets identified by dmGWAS and EW_dmGWAS were involved in cancer-related GO BP terms and KEGG pathways. Although the relationship between CL/P and cancer is still controversial in different origins, cell type, and malignancy while the co-occurrence of cancer and CL/P were greatly documented. According to a study conducted by Kobayashi et al., CL/P and cancer share common genetic variations or biological pathway alterations [12]. Based on the results of an epidemiology study in 2010, testicular cancer and melanoma rates are higher in the cancer patients with CL/P family history than those without such history [29]. Researchers from the University of Pittsburgh evaluated the cancer risk in family members of CL/P children. The results indicated that the risk ratio of cancer in CL/P individuals is 6.22 higher than that of the general population [30]. Additionally, some epidemiology studies indicated that individuals with CL/P represented a higher incidence rate of cancers in the brain, lung, and breast than normal individuals [31, 32].

Evidence of cell adhesion and cellular structure with CL/P is compelling. Our gene sets were shown to be enriched in focal adhesion, extracellular structure organization, cell-substrate adhesion, cell junction organization. This is consistent with the findings in many other publications. A literature review also highlights the importance of cell adhesion and structure in palate development [33]. Our study also suggests that genes related to CL/P are associated with the Fc gamma receptor signaling pathway and virus infection. Fc gamma receptor is a protein in cell surface involved in regulations of the immune response. Although there is no current direct evidence in the relationship between the Fc gamma receptor and CL/P, our results suggested the potential contribution of the immune response to the development of CL/P. We also observed virus infection enriched in our network module genes. A previous case-control study had found that respiratory virus infection was an important risk factor of cleft lip [34]. However, such results require further experimental validation and illustration of the causal effect of virus infection on CL/P.

Furthermore, our results showed the relationship between ErbB family and CL/P. Existing studies about ErbB family mostly focus on its relationship with solid tumors or neurodegenerative diseases. Mice with a deficiency for *Egfr*, a member of the ErbB family, exhibit facial anomalies and impaired epithelial development through a failure of secretion of matrix metalloproteinases [35–37]. These studies may serve as genetic correlation evidence of ErbB family in human CL/P.

Although our network analysis has successfully identified several candidate gene sets, there are still some limitations. One limitation of our research is that the resultant network greatly depends on the background human PPI network and GWAS signals. The non-overlap of candidate genes between the best modules for European GWAS dataset and for Asian GWAS dataset might reflect this limitation. On one hand, the dense module searching method of dmGWAS and EW_dmGWAS expanded the module by adding all the neighborhood genes within a pre-defined distance and selected the best module. The interactions in the PPI network may have a great effect on the selected modules. On the other hand, the genes with strong GWAS signals were more likely to be included in the module with high degrees of connectivity. Therefore, the top modules would be driven by these genes and the results may be biased. Although the corresponding adjustment was made to address such concern, the degree of adjustment and the impact on the final module remain unclear. Our EW_dmGWAS could partially reduce such biases because it requires both association and expression signals. Additionally, we used a one directional strategy to detect consistent signals (European data as discovery and then Asian data as evaluation) rather than a bi-directional strategy. The one directional strategy may create a bias on the results in terms of accuracy and consistency, while the bi-directional would add complexity in comparing and summarizing the results. Another limitation is that the sample size of gene co-expression data is quite small. The false positive rate would be higher when inferring edge weight according to Pearson’s correlation coefficients in cases and controls. However, the gene expression dataset we used is currently the largest such kind of data for CL/P.

## Conclusions

We presented an integrative, multi-omics study to identify novel CL/P-associated genes using our in-house network tools, dmGWAS and EW_dmGWAS, based on GWAS data, the human PPI network, and differential gene expression profiles. A total of 87 genes were consistently detected in both European and Asian ancestries in dmGWAS. In EW_dmGWAS, we identified 253 and 245 module genes associated with CL/P for European ancestry and the Asian ancestry, respectively. Functional enrichment analysis revealed that these genes were involved in cell adhesion, protein localization to the plasma membrane, the regulation of the apoptotic signaling pathway, and other pathological conditions. In addition, only a minority of genes had prior evidence in CL/P as annotated in the CleftGeneDB database. Our study highlighted nine novel CL/P candidate genes (*BRD1, CREBBP, CSK, DNM1L, LOR, PTPN18, SND1, TGS1,* and *VIM*) and 17 previously reported genes. The genetic association signals identified through our unique network analysis of GWAS pipeline provide valuable insights into the etiology of CL/P, and these new candidate genes warrant further investigation in future.

## Methods

### GWAS dataset processing

We downloaded the GWAS data from the database of Genotype and Phenotype (dbGaP) (accession number: phs000094.v1.p1). The original GWAS was conducted by the Gene Environment Association Studies (GENEVA) [11]. The CL/P cases were recruited from a number of different populations through a treatment center or population-based registry. A total of 7089 subjects consisting of case-parent trios were genotyped using the Illumina Human610_Quadv1_B array. After a series of quality control process (e.g. missing rate call, chromosome anomalies, minor allele frequency, Mendelian error, duplication error rate, and Hardy-Weinberg equilibrium filtration), the cleaned data included 2037 complete parent-offspring trios (6111 subjects) and 553,665 SNPs. We conducted the family-based association test using the transmission disequilibrium test (TDT) implemented in software plink [38, 39] for trios of Asian ancestry (1029) and trios of European ancestry (878), respectively. From the plink output, we selected the asymptotic *p*-values to compile gene-level p-values for disease association.

### Gene-based association test using pathway scoring algorithm

Gene-based p-values were compiled by combining SNP asymptotic p-values from TDT. Specifically, we mapped a SNP to a gene if it was located in the gene body or within 50 kb upstream or downstream of the gene. We used the pathway scoring algorithm, named Pascal, to calculate the gene-based p-values [40]. Pascal utilized the sums of chi-squared statistics for SNP *p*-values while controlling potential biases from gene length, SNP density, and the local Linkage Disequilibrium (LD) structure. For the TDT results using trios of European ancestry and the TDT results using trios of Asian ancestry, we used the corresponding 1000 Genomes reference panel, EUR and ASN (phase 1, release v3), respectively. In the Pascal results, we selected genes that passed the analyses (i.e., labeled with success in the status column). The gene-based p-values were then transformed to gene-based z-scores by the inverse normal distribution function.

### Human protein-protein interaction (PPI) network

We downloaded the Human Protein Reference Database (HPRD, release 9) to build the reference network, as shown previously [41]. HPRD collected experimentally validated PPIs (in vivo, in vitro, and yeast 2-hybrid) and had high reliability [42, 43]. The current version of HPRD contains a total of 9617 nodes (genes) and 39,240 edges (interactions).

### Dense module search using GWAS data (dmGWAS 2.4)

#### Single GWAS dataset

The dmGWAS version 2.4 R package was utilized to detect CL/P-associated modules and genes by superimposing GWAS signals onto the HPRD network [15]. dmGWAS implements a dense module searching (DMS) algorithm and works on the reference network with nodes weighted by gene-based z-scores. Briefly, DMS considers every gene in the reference network with GWAS p-values as a seed gene to initiate a greedy searching process. With the seed gene, DMS expands a module by examining and recruiting neighborhood genes that could improve the module score (Z_m_) by a predefined threshold, *r*. The neighborhood genes are defined as genes whose distance to genes in the module is equal to or less than a predefined distance *d*. The module score Z_m_ is calculated by using gene-level z-scores of component genes in the module. A new module score is calculated every time when new genes are added to the module. The module is finalized when all the candidate neighborhood genes fail to achieve Z_m + 1_ > Z_m_ × (1 + r), where Z_m_ is the current module score and Z_m + 1_ is the new module score should a gene be recruited into the current model. The parameter r represents the restriction on the expansion of a module. A smaller value of r (e.g., r = 0.05) would result in a large module size whereas a larger value of r (e.g., r = 0.2) may impose a too strict restriction. We used the default parameters (d = 2 and r = 0.1) in the analysis, as recommended by dmGWAS user manual [15]. The generated modules were ranked based on the normalized module score Z_n_. The top 50 modules were chosen to build the final gene subnetwork, which was visualized by Cytoscape 3.7 [44].

#### Dual GWAS datasets

The dmGWAS version 2.4 package provides a function called *dualEval* to take as input two GWAS datasets for a discovery-evaluation design. The discovery GWAS dataset would be used to generate modules, each with a module score calculated using the discovery dataset. Subsequently, these modules are evaluated using the evaluation GWAS dataset, resulting in module scores calculated using the evaluation dataset. Modules with significant scores in both datasets are considered with consistent association signals and are selected for further analyses, as previously described [13]. The initial strategy is bi-directional by treating one dataset as the discovery dataset and the other one as the evaluation dataset, and vice versa [13]. However, in our results, we found the module scores (Z_m_) showed a positive correlation when using the European subgroup as the discovery dataset and the Asian subgroup as the evaluation dataset but found no such correlation when exchanging the roles of the two datasets (Additional file 3: Figure S3). Therefore, we chose to use the European GWAS dataset as discovery dataset and employed a one directional strategy for the analysis.

### Dense module search by combining GWAS and differential co-expression (EW_dmGWAS)

The Java version of EW_dmGWAS was utilized to generate the node- and edge-weighted PPI network considering the computing efficiency [45]. The node weight is determined by the gene-based *p*-value [19]. The edge weight is inferred by the change degree of gene expression between case and control samples. The module score is determined by node weight and edge weight and is balanced through the parameter λ [19]. λ ranges between 0 and 1 to adjust the contribution from edge weight on the module score.

We downloaded a gene expression dataset from Gene Expression Omnibus (GEO, accession ID: GSE42589) that were conducted using dental pulp stem cell samples of nonsyndromic CL/P patients. The platform was Affymetrix Human Gene 1.0 ST Array. The original file contained expression data of 33,297 probe sets of 7 case and 6 control samples. Robust multi-array average (RMA) and quantile normalization were conducted. For genes with multiple probe sets, we used the average expression of these probe sets for the gene. Finally, we obtained gene expression for 20,358 genes and they were imported into EW_dmGWAS for edge weights.

### Functional enrichment analysis of module genes

We used WebGestalt [46] for functional enrichment analysis of the candidate genes residing in the subnetworks identified by dmGWAS and EW_dmGWAS. The Gene Ontology (GO) and Kyoto Encyclopedia of Genes and Genomes (KEGG) functional databases were used. Multiple testing correction was controlled by the Bonferroni method [47]. The minimum number of genes for a category was set to 10 with the maximum number set to 500 [48, 49]. The enriched categories were identified based on the adjusted p-value threshold of 0.05. Affinity propagation, one method of redundancy reduction, was used as a post-processing step to identify the most significant and representative sets after clustering gene sets based on the Jaccard index [46].

### Literature mining of CL/P candidate genes

Our in-house database, CleftGeneDB [22], was used to curate CL/P candidate genes from literature mining. Specifically, we systematically performed literature mining of CL/P genes in humans and mice followed manual curation by domain experts. The data deposited in CleftGeneDB contained 194 human CL/P genes and 272 mouse CL/P genes. The mouse mutation genes were mapped to the homologous human genes. These gene sets were compared with the genes identified through dm_GWAS and EW_dmGWAS.

## Supplementary information

**Additional file 1:** **Figure S1.** Manhattan plots of gene-based *p*-values generated by Pascal for the European ancestry (A) and Asian ancestry (B)

**Additional file 2:** **Figure S2.** Subnetworks of module genes identified by dmGWAS for the European ancestry (A-B) and Asian ancestry (C-D)

**Additional file 3:** **Figure S3.** Distribution of module scores (Z_m_) from two GWAS datasets

## Data Availability

All the data used in this study are from public sources cited in our reference list. Also, Additional files, which may be needed to reproduce the results presented in the manuscript, are made available as Additional files.

## Abbreviations

CL/P: Cleft lip with or without cleft palate
dbGaP: Database of Genotype and Phenotype
dmGWAS: Dense module search for Genome-Wide Association Studies
EW_dmGWAS: Edge-Weighted dense module search for Genome-Wide Association Studies
GO: Gene Ontology
GSEA: Gene Set Enrichment Analysis
GWAS: Genome-Wide Association Studies
HPRD: Human Protein Reference Database
KEGG: Kyoto Encyclopedia of Genes and Genomes
Pascal: Pathway scoring algorithm
PPI: Protein-protein interaction
