# Supplementary figures and images for: An integrative, genomic, transcriptomic and network-assisted study to identify genes associated with human cleft lip with or without cleft palate
Source: BMC Med Genomics. 2020 Apr 3;13(Suppl 5):39. doi: 10.1186/s12920-020-0675-4 (PMC7118807; doi:10.1186/s12920-020-0675-4)

A

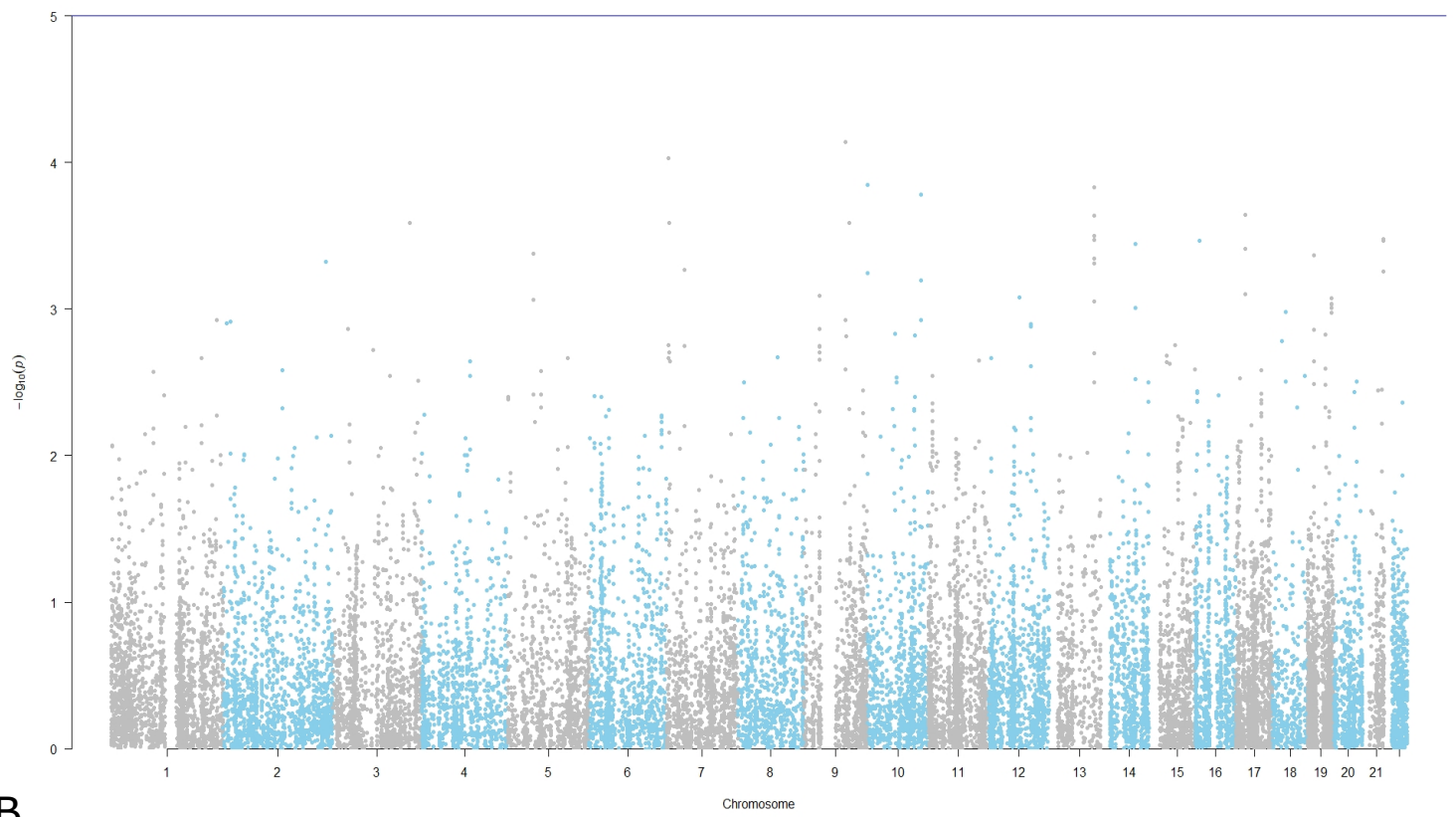

B

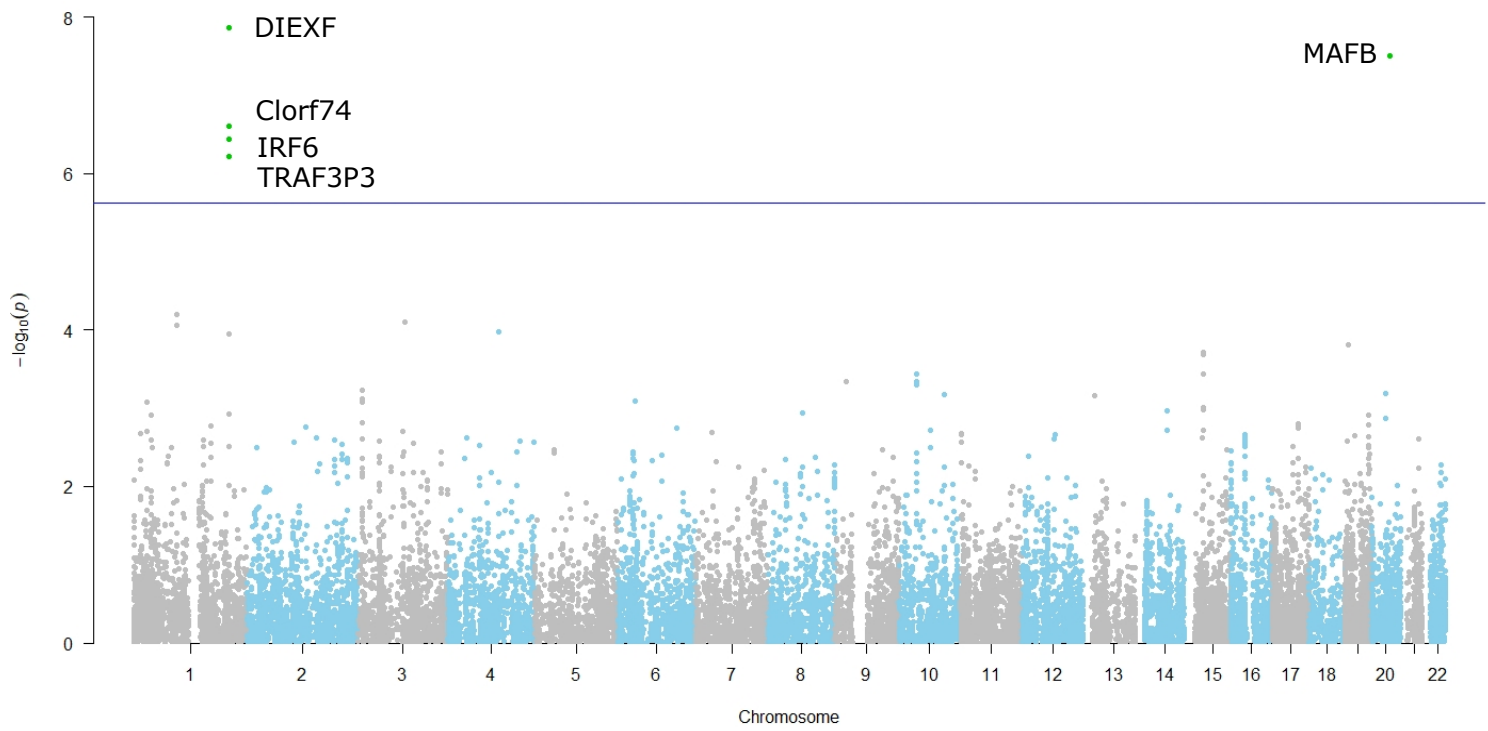

Supplement: Supplementary file 1 — Additional file 1: Figure S1. Manhattan plots of gene-based p-values generated by Pascal for the European ancestry (A) and Asian ancestry (B) [file 12920_2020_675_MOESM1_ESM.pdf]

A

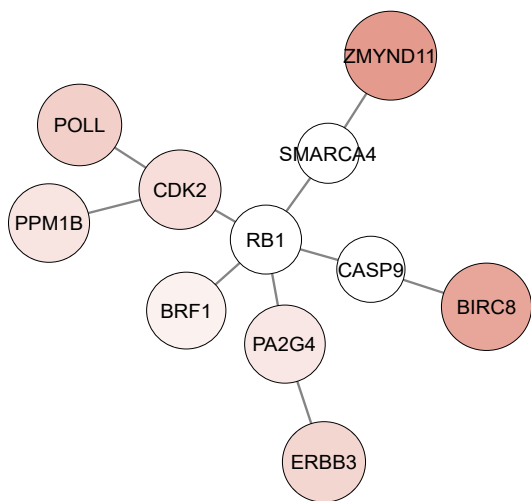

C

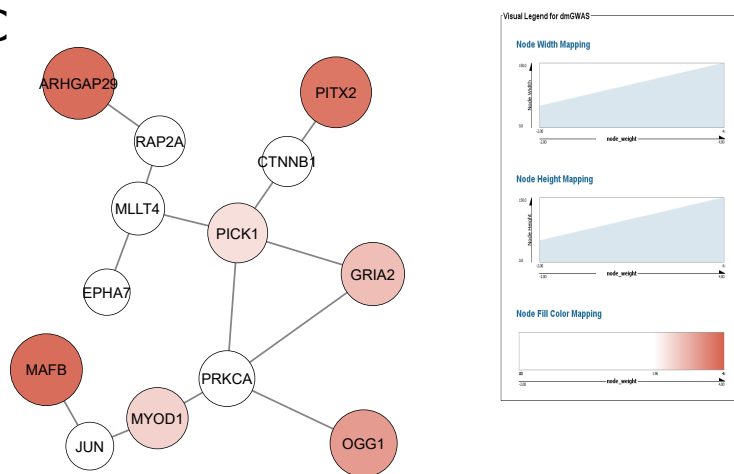

B

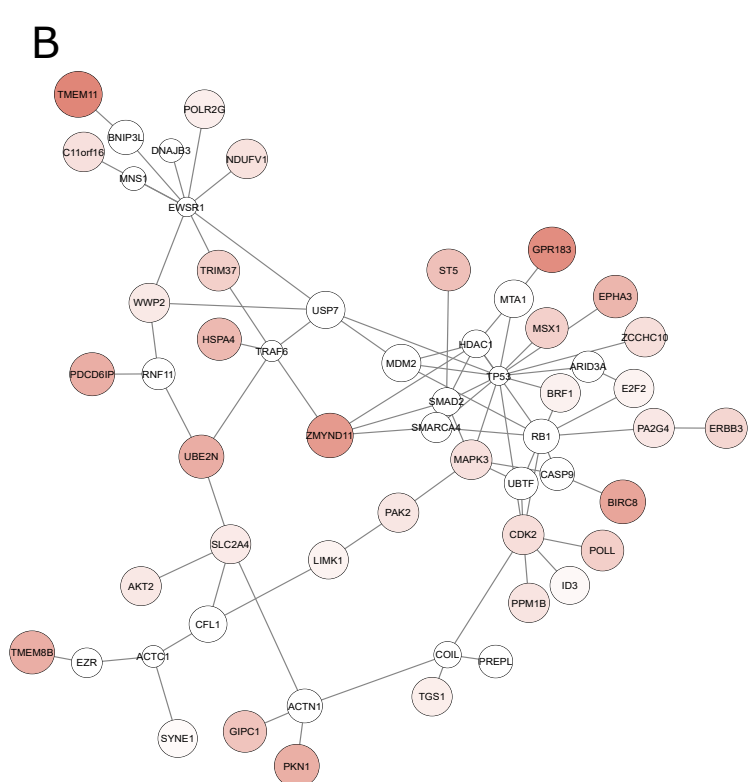

D

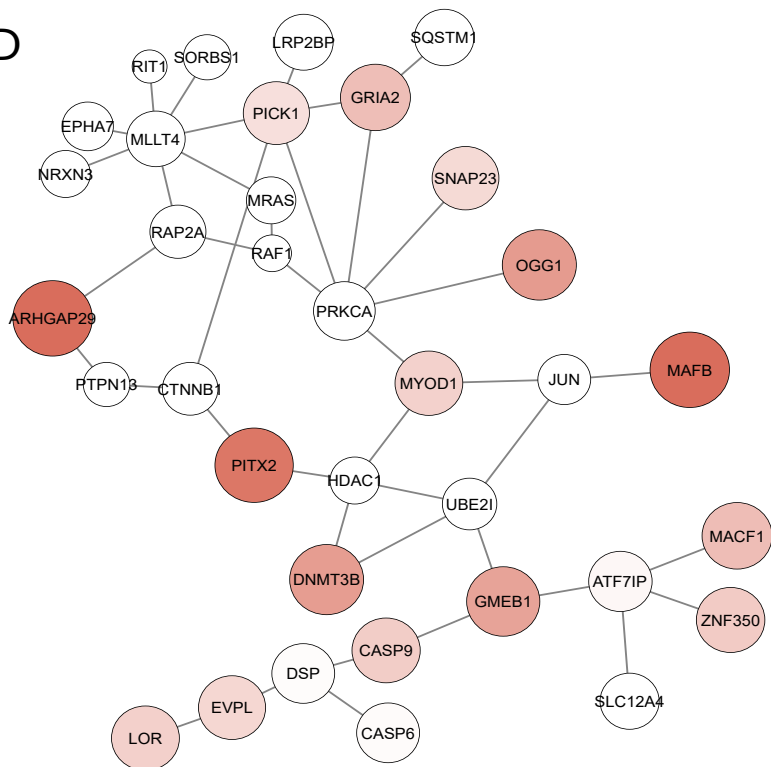

Supplement: Supplementary file 2 — Additional file 2: Figure S2. Subnetworks of module genes identified by dmGWAS for the European ancestry (A-B) and Asian ancestry (C-D) [file 12920_2020_675_MOESM2_ESM.pdf]

A

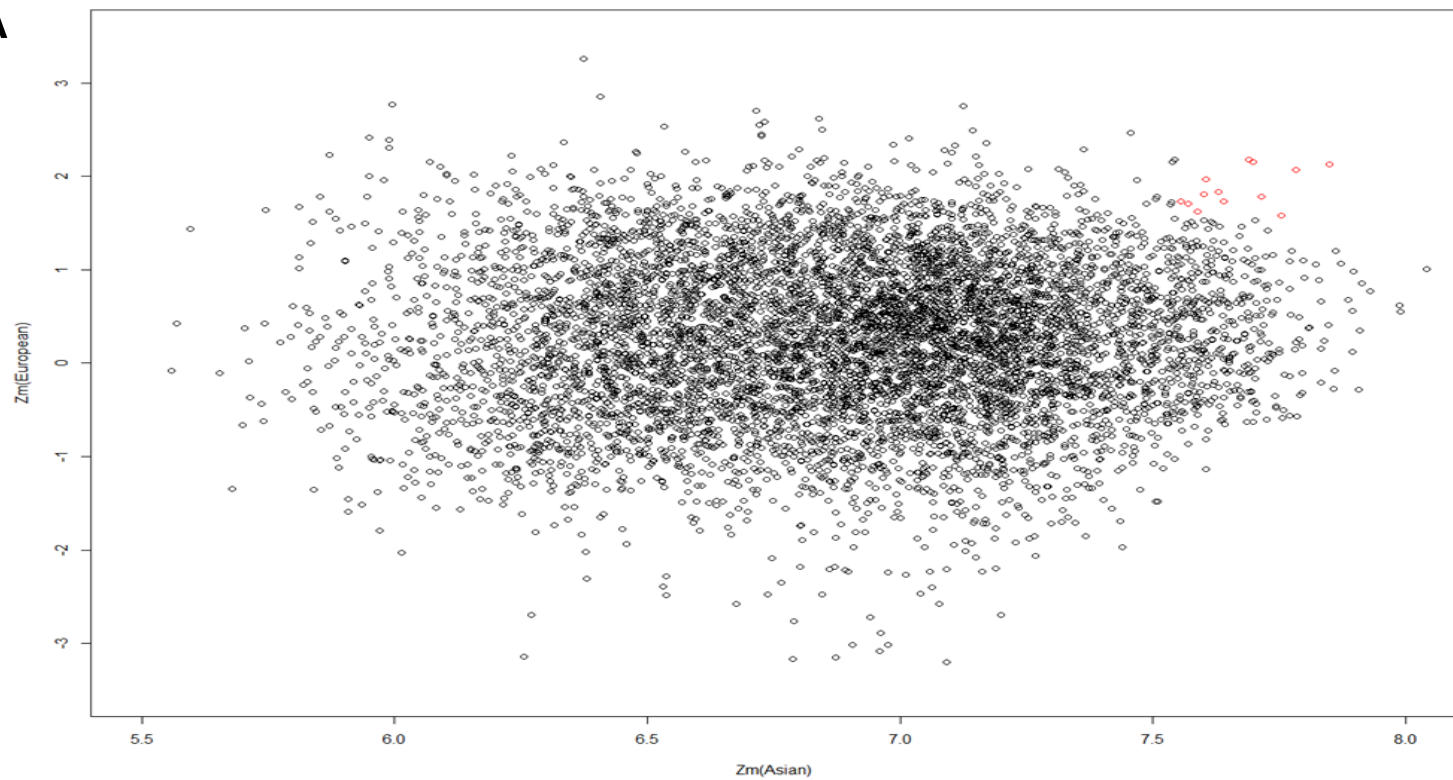

B

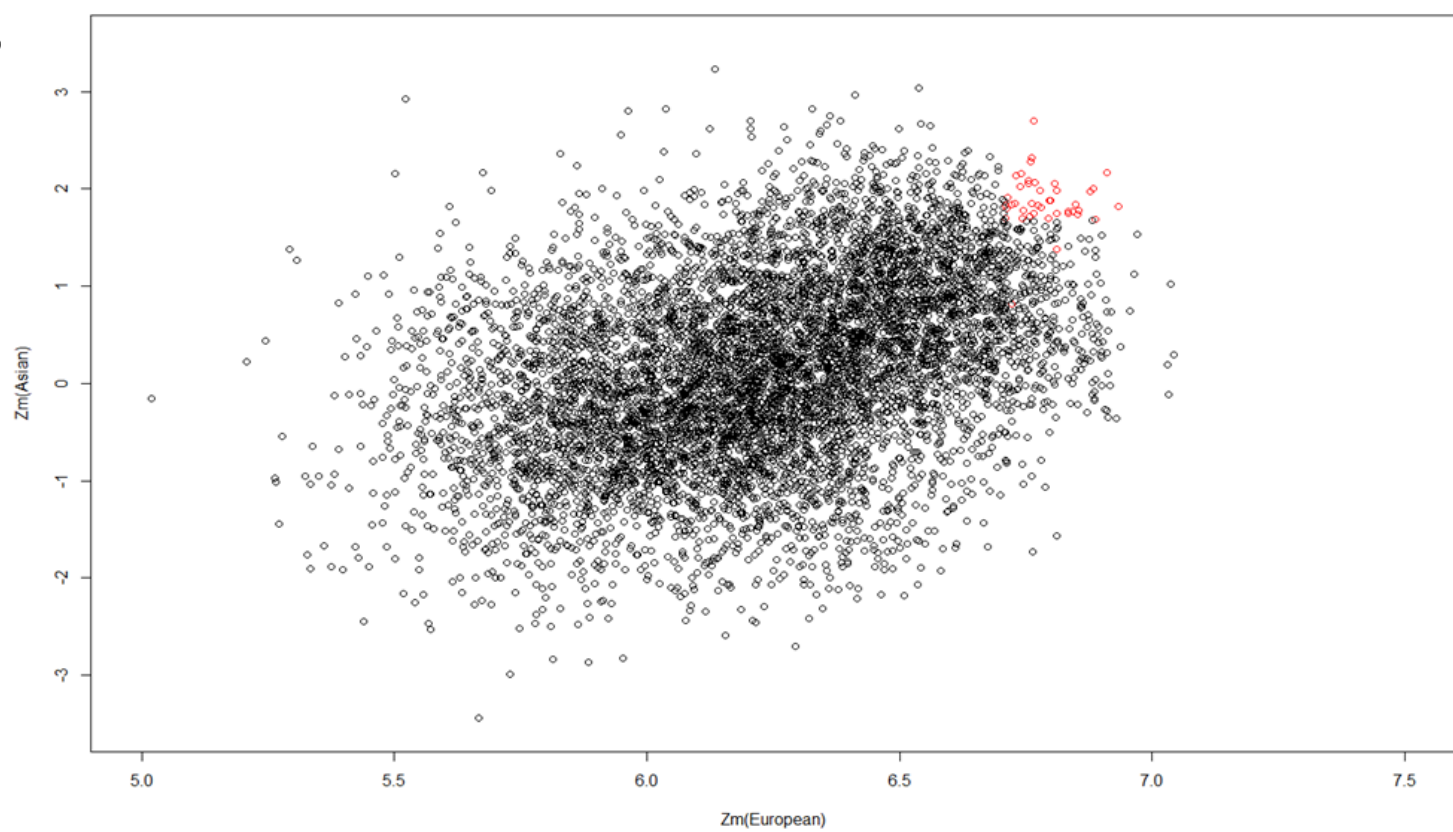

Supplement: Supplementary file 3 — Additional file 3: Figure S3. Distribution of module scores (Zm) from two GWAS datasets [file 12920_2020_675_MOESM3_ESM.pdf]
